# Severe Abdominal Pain

**Published:** 1903-06-13

**Authors:** J. M. Fortescue-Brickdale

**Affiliations:** Registrar and Pathologist to the Children's Hospital, Bristol


### Hospital Clinics and Medical Progress.

### SEVERE ABDOMINAL PAIN.

By J. M. Fortescue-Brickdale, M.A., M.D. Oxon., Registrar and Pathologist to the Children's Hospital, Bristol.

The acute or even sudden occurrence of severe abdominal pain is frequently the reason for which medical advice is sought, and the practical question which generally calls for an answer in these cases is whether or no immediate operative measures are necessary. Though it may be laid down as a good general rule that all doubtful cases should at once be submitted to a surgeon, this may not always be feasible; and anyhow it is the duty of the general practitioner to attempt to make a diagnosis for himself. The practical issue can only satisfactorily be decided after every detail of the case has been thoroughly observed and sifted; a proceeding which will invariably increase the practitioner's knowledge and judgment, and may save the patient much unnecessary inconvenience and expense. There is no excuse for "expectant treatment" in these cases, but in all careful investigation and thought are most necessary.

The principal causes of severe abdominal pain are:—

1. Perforation of an abdominal viscus.
2. Obstruction or strangulation of the intestine.
3. Certain pelvic conditions in women, *e.g.* twisting of an ovarian pedicle, rupture of a pyosalpynx or ectopic fœtation.
4. Acute hæmorrhagic pancreatitis.
5. Hæmorrhage into the peritoneum, as from ruptured aneurysm.
6. Intestinal colic; biliary or renal colic.
7. Acute gastritis, *e.g.*, from irritant poisoning.
8. Visceral crises in tabes dorsalis.
9. Diaphragmatic pleurisy.

The early symptoms which may accompany any of these conditions are extreme pain and collapse, vomiting, shallow respiration, a small rapid pulse and an anxious expression of countenance. This symptom group (the "peritonism" of Grübler) is of course liable to individual variations. Its modification or incompleteness may indicate that no serious abdominal lesion has occurred; on the other hand, considerable divergence from the typical clinical picture cannot of itself be taken as evidence of the comparatively harmless nature of the case.

The first five conditions on the list may be considered as requiring immediate laparotomy; in the rest surgical measures are either unnecessary or may safely be postponed. A final judgment can only be attained by a synthesis of all the conditions of the case, and this in turn will depend upon a careful preliminary analysis. The chief points to be remembered in this analysis will now be separately discussed.

1. *Expression and Position.*—The abdominal expression is very characteristic, and is usually well marked in the severer class of cases. In intestinal colic it is rare to find the patient exhibiting the extremely drawn, pinched, anxious expression which is seen in actual perforation or strangulation. On the other hand the absence of this appearance in early cases is not absolute proof that no grave lesion has occurred. The patient when in paroxysms of pain may roll about, the knees are usually drawn up to relax the pressure of the abdominal muscles; in diaphragmatic pleurisy, however, the patient is said to be generally found sitting up in bed, bending forward, whilst in intestinal colic he may walk about pressing the abdomen in with his hands or leaning over the back of a chair in order to ease the pain.

2. *Pain.*—Comparatively little importance can be attached to the apparent severity of the pain. Diffuse pain is more common in perforation, localised pain in colic or gastritis. The localisation of the pain, whilst always suggestive, is not by any means invariably conclusive. Early pain in many conditions is often referred to the umbilicus. In gastric or duodenal ulcer, in diaphragmatic pleurisy, gastric "crises," and simple gastritis it may be felt mostly over the upper part of the abdomen. In visceral "crises" the pain may be general. On the other hand, in gastric ulcer the pain is sometimes most intense in the right iliac fossa, simulating appendicitis. Pain on the left side radiating into the penis, scrotum, or inner side of the thigh is naturally most suggestive of renal colic, but on the right side this radi[illegible]ing pain is sometimes found to be due to appendicular disease. In gall-stone colic the pain is most intense in the right hypochondrium, as a rule, and may radiate backwards and upwards into the thorax and right shoulder. In intestinal colic pain is often relieved by pressure, which is a very rare occurrence in other conditions; marked abdominal hyperæsthesia has, however, been noticed in intestinal colic, especially in nervous or hysterical subjects. It should be remembered that some cases of influenza begin with severe abdominal pain.

3. *Vomiting.*—The most severe and persistent vomiting occurs perhaps in strangulation of the intestine either by hernia or otherwise. Fæcal matter may eventually be ejected. In perforated gastric ulcer there may be an initial emesis, but this is generally slight and not long continued, a point of distinction sometimes made between this condition and duodenal ulcer. Vomiting in gastric "crises" may be completely absent, and may be only a late symptom in obstruction of the bowel low down in its course. In appendicular disease (and perhaps in some other conditions) vomiting may be slight if constipation is absent.

4. *Temperature and Pulse.*—A raised temperature is sometimes observed in cases where perforation has taken place, but is usually in these cases (and in strangulation of the gut) a sign of the onset of peritonitis. A rigor with pyrexia is not infrequently observed to usher in an attack of renal or biliary colic, and may serve to distinguish the latter from gastritis. High temperatures are noted in diaphragmatic pleurisy. Rigors are rare in conditions precedent to peritonitis. The small, thready, rapid pulse of collapse is often a valuable sign, indicating a severe intestinal lesion; but it is often present also in biliary and renal colic. In intestinal colic it may occur, but here the pulse is generally fuller and slower.

(*To be Continued.*)

